# Quantitative Bioluminescent Imaging of AHR Activity Using a Thermostable Variant of the Fungal Luciferase nnLuz

**DOI:** 10.3390/ijms27219586

**Published:** 2026-10-27

**Authors:** Hannah J. Gleneadie, Nicolas Veland, Nadezhda M. Markina, Alessandro Sardini, Anastasia A. Fadeeva, Zoe Webster, Feng Gao, Matthias Merkenschlager, Amanda G. Fisher, Karen S. Sarkisyan

**Affiliations:** 1 Epigenetic Memory Group, MRC Laboratory of Medical Sciences, Imperial College London, Hammersmith Hospital Campus, Du Cane Road, London W12 0HS, UK; nicolas.veland@crick.ac.uk (N.V.); amanda.fisher@bioch.ox.ac.uk (A.G.F.); 2 Planta LLC, Skolkovo Innovation Center, Bolshoy Boulevard 42, Building 1, 335, Moscow 121205, Russia; nadya.markina@planta.bio; 3 Shemyakin-Ovchinnikov Institute of Bioorganic Chemistry, Russian Academy of Sciences, Moscow 117997, Russia; 4 Whole Animal Physiology and Imaging, MRC Laboratory of Medical Sciences, Imperial College London, Hammersmith Hospital Campus, Du Cane Road, London W12 0HS, UK; a.sardini@lms.mrc.ac.uk; 5 Transgenics & Embryonic Stem Cell Facility, MRC Laboratory of Medical Sciences, Imperial College London, Hammersmith Hospital Campus, Du Cane Road, London W12 0HS, UK; zoe.webster@lms.mrc.ac.uk; 6 Synthetic Biology Group, MRC Laboratory of Medical Sciences, Imperial College London, Hammersmith Hospital Campus, Du Cane Road, London W12 0HS, UK; f.gao21@lms.mrc.ac.uk; 7 Lymphocyte Development Group, MRC Laboratory of Medical Sciences, Imperial College London, Hammersmith Hospital Campus, Du Cane Road, London W12 0HS, UK; matthias.merkenschlager@lms.mrc.ac.uk; 8 Department of Biochemistry, University of Oxford, Oxford OX1 3QU, UK; 9 Institute of Clinical Sciences, Faculty of Medicine and Imperial College Centre for Engineering Biology, Imperial College London, London W12 0HS, UK

**Keywords:** bioluminescence, fungal luciferase, aryl hydrocarbon receptor (AHR), Cyp1a1, endogenous reporter, autoluminescence

## Abstract

Bioluminescent reporters are important tools in biomedical research. Luciferase enzymes, expressed as part of transgenes or inserted into endogenous genomes, can be used to visualise molecular events with minimally invasive experiments. However, the reaction requires addition of the substrate, luciferin. Organisms with self-sustained luminescence that both express the luciferase and produce the luciferin would allow continuous, convenient and affordable acquisition of physiological information. The *Neonothopanus nambi* fungal bioluminescence pathway has been harnessed in this way to produce autoluminescent plants. However, progress into mammalian models has been hindered by temperature sensitivity of the wild-type fungal luciferase (nnLuz). We tested the efficacy of a thermostable variant of nnLuz (nnLuz_v3) in vivo at physiologically relevant levels, by generating a reporter of endogenous *Cyp1a1* expression as a measure of aryl hydrocarbon receptor (AHR) activation. Previously published *Cyp1a1-Fluc* mice allowed a comparison between the novel luciferase nnLuz_v3 and the established firefly luciferase (Fluc). In *Cyp1a1-nnLuz_v3* mice, the bioluminescence signal was sufficiently stable to enable well-resolved luminescence images to be captured on standard in vivo imaging equipment and consumer-grade cameras. These results derisk the use of nnLuz_v3 as an in vivo molecular reporter whilst providing a novel tool for AHR drug discovery and research.

## 1. Introduction

The use of reporter genes to visualise molecular events is an important tool in biological research, with light being the most commonly used modality. Plants, animals and cellular systems are modified to express fluorescent or luminescent reporters upon activation of a regulatory region or a gene of interest. In animals, fluorescence-based reporters become largely unusable in vivo due to autofluorescence of tissues and poor delivery of excitation light. In contrast, bioluminescent reporters possess the advantage of virtually no background signal [1,2]. The enzyme luciferase catalyses the luminescence reaction, oxygenating the substate luciferin and producing light as a biproduct. Hundreds of examples of luminescence have been identified throughout the natural world, with a number of original and modified luciferase/luciferin pairs used regularly in biomedical research [3,4]. However, these rely on the exogenous addition of the luciferin substrate, making imaging invasive, discontinuous, expensive and subject to uneven tissue distribution. Therefore, for a number of in vivo applications, the ultimate light-based molecular reporter could be self-sustained luminescence, where both the luminescence substrate and the luciferase enzyme are produced in a single organism.

**Figure 1:**
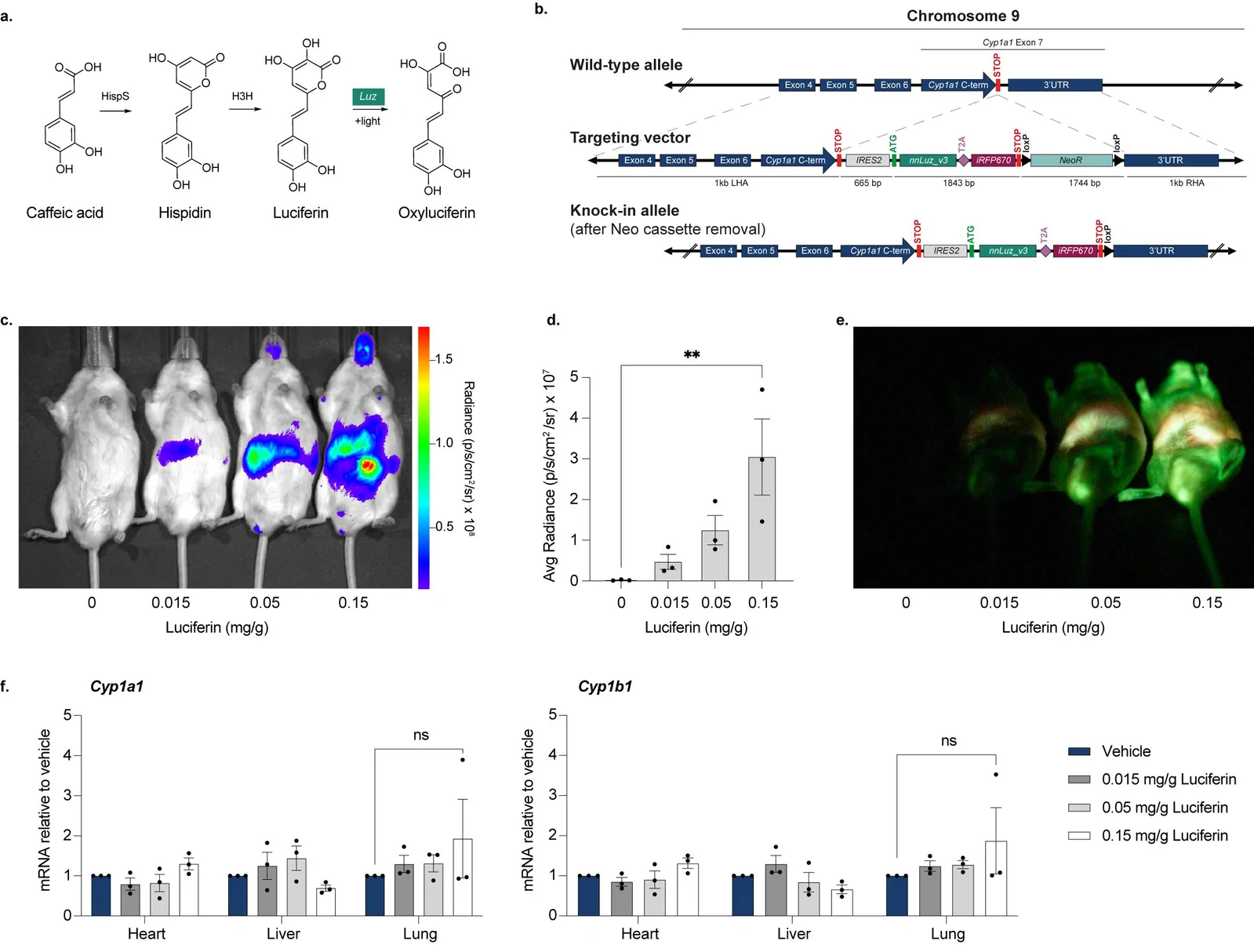
Generation and characterisation of endogenous bioluminescent reporter of AHR activation using fungal luciferase *nnluz_v3.* (**a**) Schematic outlining the fungal luciferin pathway. Hispidin synthase (HispS) converts caffeic acid into hispidin, which is then hydroxylated by H3H into 3-hydroxyhispidin (fungal luciferin). The luciferase enzyme (Luz) converts luciferin into oxyluciferin and produces light [6]. (**b**) Schematic outlining the engineered *Cyp1a1* locus in *Cyp1a1-nnLuz_v3* mice and mouse embryonic stem cells (mESCs). *nnLuz_v3* and *iRFP670* were inserted before the 3′UTR of endogenous *Cyp1a1*. An *IRES2* sequence separates the reporter genes from the *Cyp1a1* C-terminal and a *T2A* sequence separates the two reporter genes. (**c**) Representative bioluminescent image of *Cyp1a1-nnLuz_v3* adult mice 5 h post-intraperitoneal (IP) injection with 3-mC. IP injection of fungal luciferin at concentrations of 0, 0.015, 0.05, and 0.15 mg/g show dose-dependent luminescence. (**d**) Quantification of (**c**). Graph shows mean average radiance of whole mice (*n* = 3) ± SEM. A one-way ANOVA compares vehicle with luciferin-injected animals with Dunnett’s multiple comparison test. ** *p* < 0.01. (**e**) The same animals from (**c**) imaged using a Sony Alpha 1 camera with SEL50F12GM lens, ISO 800 and exposure of 300 secs. (**f**) Heart, liver, and lung tissues dissected from *Cyp1a1-nnLuz_v3* mice 1 h after fungal luciferin injection. Relative *Cyp1a1* and *Cyp1b1* mRNA levels as a measure of AHR activation. *Cyp1a1* and *Cyp1b1* mRNA levels were normalised to housekeeping gene *Tbp* and are shown relative to vehicle-treated control. Bars show mean (*n* = 3) ± SEM with a two-way ANOVA to compare vehicle- to luciferin-treated samples and Dunnett’s test to correct for multiple comparisons. ns = not significant.

Only two luciferin biosynthesis pathways have been fully deciphered: the bacterial (lux) pathway [5] and the fungal (luz) pathway [6]. Constitutive expression of the complete bacterial pathway has been used to generate autoluminescent mice [7], which have been further adapted for cellular-scale autoluminescent imaging of neurons [8]. However, whilst the expression of the complete fungal bioluminescence pathway can generate autoluminescent plants and fungi [9,10,11], it has not yet been used in warm-blooded animals. The *Neonothopanus nambi* bioluminescence pathway is a short cycle within fungal phenylpropanoid metabolism, catalysed by four enzymes, with the light-emitting reaction performed by the luciferase, nnLuz (Figure 1a) [11]. Wild-type nnLuz displays poor activity at temperatures above 30 °C, limiting its use in mammalian cells [6]. To address this, we previously showed that variants carrying three substitutions, T99P, T192S and A199P (nnLuz_v3), outperformed wild-type controls when transiently expressed in mammalian cells at 37 °C [10]. However, whether this improvement would translate into transgenic animal models was unclear. Here, we tested the efficacy of this thermostable nnLuz variant in mice at physiologically relevant expression levels using aryl hydrocarbon receptor (AHR) activation as a model.

The aryl hydrocarbon receptor (AHR) is a soluble transcription factor that recognises numerous endogenous and environmental ligands, adjusting cellular physiology to maintain tissue homeostasis [12]. Activation of one of its downstream effectors, the cytochrome P450 protein Cyp1a1, provides a reliable measure of AHR activity [13,14]. The pathway is dynamic and reversible, making it an ideal system to test novel reporter systems, therefore, we developed *Cyp1a1-nnLuz_v3* mice where *nnLuz_v3* was targeted to the 3′UTR of endogenous *Cyp1a1*. Here, we compared *Cyp1a1-nnLuz_v3* mice with a previously described *Cyp1a1-Fluc* reporter mouse line [15] and found comparable luminescence intensity and signal distribution. We examined the emission spectrum and substrate kinetics of the two luciferase/luciferin pairs and found that the in vivo emission of nnLuz_v3 could be visualised on consumer-grade cameras. These results derisk the use of the nnLuz_v3 in vivo, suggesting that the fungal luciferase can be used as a molecular reporter in warm-blooded animals, encouraging further optimisation of the fungal luciferin biosynthesis pathway for use in mammalian cells.

## 2. Results

To determine whether nnLuz_v3 is suitable for in vivo bioluminescence imaging at physiologically relevant levels, we generated an nnLuz_v3 reporter of endogenous AHR activity. The *nnLuz_v3* gene and the far-red fluorescent protein *iRFP670* (included to validate entry of transgenes) were inserted into the 3′UTR of endogenous *Cyp1a1* in the mouse genome. Reporter genes were driven by IRES2 and separated by a T2A site (Figure 1b); therefore, distinct Cyp1a1, iRFP670 and nnLuz_v3 polypeptides were generated from a single RNA transcript, whilst the function of the targeted *Cyp1a1* allele was expected to be unaffected. Integration of *IRES2-nnLuz_v3-T2A-iRFP670* cassette into mESCs resulted in multiple knock-in clones that luminesced upon treatment with the AHR ligand 6-formylindolo [3,2-β]carbazole (FICZ) (clones A4 and C12 are shown as examples in Supplementary Figure S1a, luminescence signal in blue/green, quantified in b). Clone A4 was used to generate transgenic mice. Transgenic animals appeared to develop normally and showed no apparent phenotypic differences compared to wild-type controls. Pre-treatment with long-acting AHR agonist 3-methylcholanthrene (3-mC) led to development of robust luminescence signal, with brightness dependent on concentration of intraperitoneally (IP) injected luciferin (Figure 1c, quantified in d). Encouragingly, brightness was sufficient for imaging on consumer-grade cameras with 50-megapixel resolution (Figure 1e). Within an hour of fungal luciferin injection, animals displayed no visible phenotypic changes and, reassuringly, no change in tissue mRNA levels of AHR response genes *Cyp1a1* or *Cyp1b1*, suggesting the luciferin did not activate or repress canonical AHR signalling (Figure 1f). The slight, non-significant increase in expression of AHR response genes at 0.15 mg/g in the lungs suggests that this concentration of luciferin should be used as the upper limit for future experiments. From these experiments we cannot discount delayed AHR activation or toxicity associated with repeated luciferin exposure; therefore, these would need to be determined before the *Cyp1a1-nnLuz_v3* animals are used in longitudinal studies.

**Figure 2:**
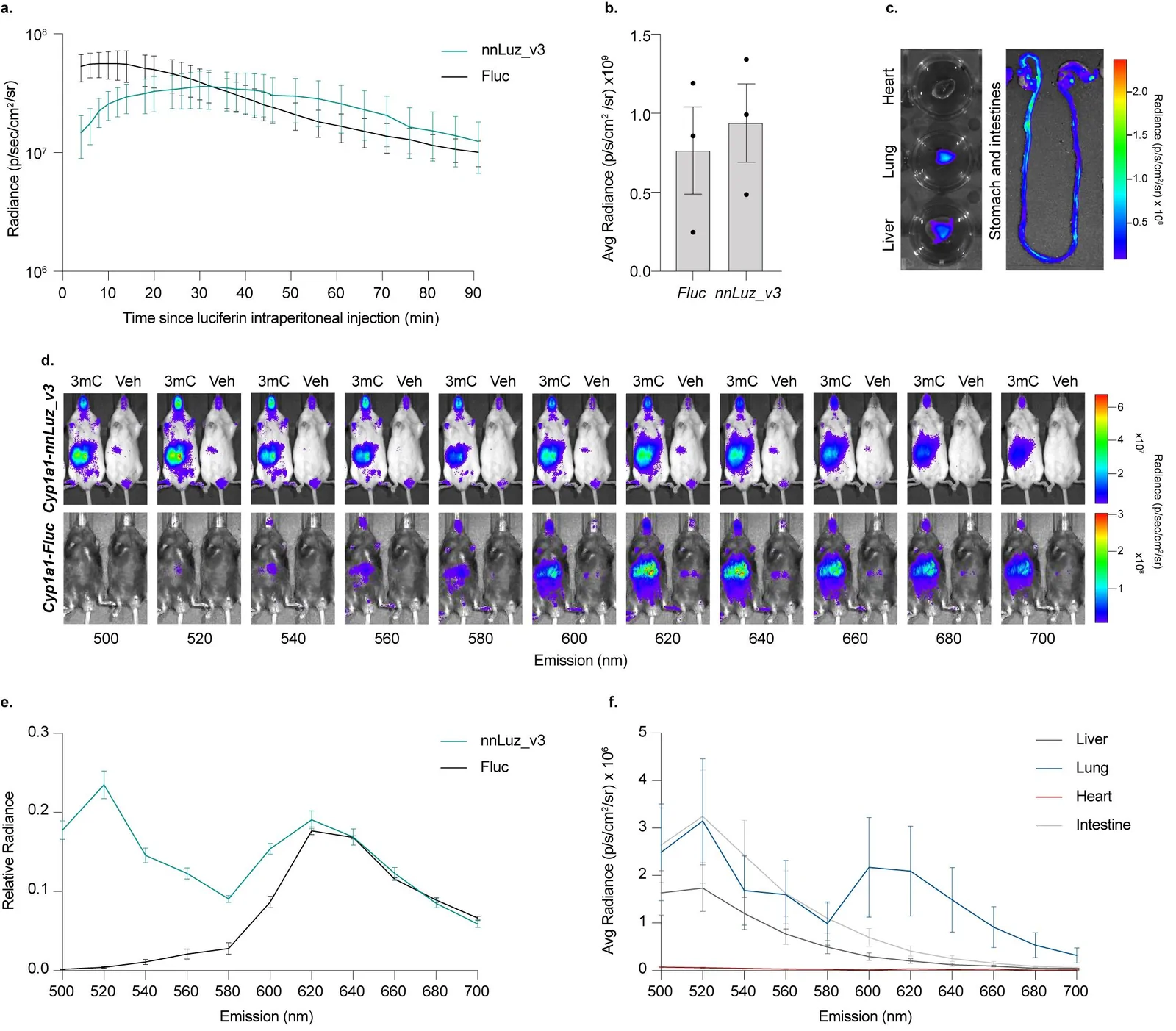
Comparison between nnLuz_v3 and Fluc as in vivo reporters of AHR activity. (**a**) Quantification of luminescence emitted from *Cyp1a1-nnLuz_v3* (teal) and *Cyp1a1-Fluc* (black) mice imaged repeatedly across a 90 min time period following IP injection with 0.15 mg/g fungal luciferin or D-luciferin, respectively. Mice were IP injected with 3-mC 5 h prior to the start of imaging. Graph shows mean whole-body radiance (*n* = 3) for each time point ± SEM. (**b**) Total radiance emitted by each genotype across the period shown in (**a**). Graph shows mean (*n* = 3) ±SEM. (**c**) Representative bioluminescence images of tissues dissected from *Cyp1a1-nnLuz_v3* mice 30 min after IP injection of 0.15 mg/g fungal luciferin. (**d**,**e**) *Cyp1a1-nnLuz_v3* (nnLuz_v3) and *Cyp1a1-Fluc* (Fluc) adult mice were IP injected with 0.15 mg/g fungal luciferin or D-luciferin, respectively, and imaged using 20 nm bandpass emission filters across a 500–700 nm range. Mice were IP injected with vehicle or 3-mC 5 h prior to luciferin exposure; for each genotype and emission filter, representative images of vehicle- and 3-mC-treated mice are shown in (**d**) and quantification of whole-body radiance in (**e**). For each wavelength, whole-body radiance is normalised against corresponding open-filter value. Graph shows mean (*n* = 3) ± SEM. (**f**). Tissues were dissected from *Cyp1a1-nnLuz_v3* mice after IP injection with fungal luciferin. Bioluminescence imaging was performed using 20 nm bandpass emission filters across a 500–700 nm range. Graphs show quantification of average radiance (*n* = 3) ± SEM.

Previously, we described the generation of a similar reporter line, where the firefly luciferase (*Fluc*) gene is inserted into the 3′UTR of endogenous *Cyp1a1*, separated from the targeted allele by a T2A site [15]. This model (*Cyp1a1-Fluc*) provides an opportunity to compare the two luciferase/luciferin systems. Following IP injection of the luciferin substrates, Fluc luminescence peaks 15 min post-injection then deteriorates, whilst nnLuz_v3 luminescence is slower to peak but remains stronger for longer (Figure 2a). Indeed, half-life analysis of these curves suggests that while D-Luc has a t_1/2_ of 30.7 min, the t_1/2_ for fungal luciferin is almost 2 h (116.8 min). This likely demonstrates differences between the uptake and retention of the two substrates. When the total radiance emitted across the 90 min period is compared, Fluc and nnLuz_v3 show comparable levels (Figure 2b). Therefore, whilst we cannot make a direct comparison between the two luciferases due to the distinct properties of the two substrates, the comparable radiance levels suggest that nnLuz_v3 is suitable for use as an endogenous reporter in vivo. Moreover, ex vivo imaging of tissues from *Cyp1a1-nnLuz_v3* animals show strong luminescence emitted from the lungs, liver and intestine following 3-mC exposure (Figure 2c), corresponding to equivalent luminescence and mRNA results obtained previously in *Cyp1a1-Fluc* mice [15].

Fluc emits red light in vivo [16], with the peak of emission around 620 nm in *Cyp1a1-Fluc* mice (Figure 2d, lower panels, and quantified in e, black line). Interestingly, whilst bioluminescence from nnLuz peaks at 520 nm in fungi, in *Cyp1a1-nnLuz_v3* mice, luminescence peaked at 520 nm but had an additional, smaller peak at ~620 nm (Figure 2d, upper panels, and quantified in e, teal line). Ex vivo analysis of luminescence of different *Cyp1a1*-*nnLuz_v3* organs showed that most tissues emitted green light, with the exception of the lungs, which exhibited the 520/620 nm double-peaked spectrum (Figure 2f). We detected a similar result in a previously characterised green click beetle luciferase-expressing mouse line [17] (Supplementary Figure S2a), whilst all *Cyp1a1*-*Fluc* tissues exhibited a single peak of emission (Supplementary Figure S2b). This suggests that the double-peaked spectrum is not specific to the chemistry of fungal bioluminescence nor related to the expression of *Cyp1a1* but is likely an artefact of green-emitting luciferase enzymes. This could be attributed to absorption of green light by haemoglobin, which is known to interfere with the spectrum of green-emitting luciferase enzymes in vivo and is most highly abundant in the lung [18,19].

We next investigated whether fungal luciferin is orally bioavailable, a feature that could enable minimally invasive experiments with animals expressing nnLuz_v3. Mice fed fungal luciferin by oral gavage showed abdominal luminescence signal from as early as 8 min post-ingestion (Supplementary Figure S3a). However, the signal detected following the oral delivery of luciferin differed both in intensity and distribution from that of IP delivery. Following oral gavage, luminescence was primarily detected in the intestines, with very low signal detected in the lungs, heart or liver (Supplementary Figure S3b,c), likely due to luciferin retention in the digestive tract. While short-term exposure to fungal luciferin does not appear to be toxic nor activate canonical AHR signalling, it is unknown whether this accumulation of luciferin would be deleterious to the gut epithelium. This highlights the potential benefits of an autoluminescent model where the luciferin substrate is produced constitutively, and the luciferase enzyme is used as a reporter gene. Here, direct physiological information could be obtained through simple, cheap and non-invasive experiments. Towards this, we began preliminary work optimising the fungal luciferin biosynthesis pathway to function in mammalian cells. We found that, in vitro, HEK293T cells provided the brightest bioluminescence signal; therefore, these were used as a model for these experiments. Measurable bioluminescence was detected from HEK293T cells transfected with all members of the fungal luciferin biosynthesis pathway (Supplementary Figure S3d–f; note that in these experiments, we used a slightly brighter version of the luciferase, nnLuz_v4 [10]). However, luminescence was still ~1000 fold lower compared to cells with exogenously added luciferin and at a level not compatible with in vivo imaging. Indeed, addition of all pathway intermediates (caffeic acid, hispidin, oxyluciferin) (Supplementary Figure S3d) significantly increased the signal (Supplementary Figure S3e). This suggests the pathway can be further optimised in mammalian cells, with gene dosage experiments indicating that hispidin synthase (HispS) is the limiting step (Supplementary Figure S3f).

## 3. Discussion

Initially discovered as a key player in the xenobiotic response [20], AHR has since been found to respond to a vast array of environmental, dietary and microbial stimuli [12,21]. In the absence of a ligand, the receptor is sequestered in the cytosol as part of a multiprotein complex. Upon ligand binding, AHR dissociates from this complex and translocates to the nucleus where it dimerises with AHR nuclear translocator (ARNT) and activates the expression of target genes [12,21]. Among these target genes is *Ahrr*, encoding the Aryl hydrocarbon receptor repressor, which targets nuclear AHR for degradation [12]. This is one of a number of ways AHR activity is dynamically regulated [21]. The AHR pathway has been associated with numerous diseases, with AHR inhibitors already in clinical trials for solid tumours [12,22,23]. Therefore, models that non-invasively report on AHR activity have potential for therapeutic development. Previously, we described the generation of an in vivo reporter of AHR activity, where *Fluc* was inserted into the endogenous *Cyp1a1* gene [15]. Here, we developed a *Cyp1a1* reporter mouse using the novel luciferase nnLuz_v3. While both models non-invasively report on AHR activity, *Cyp1a1-nnLuz_v3* animals have three potential advantages over *Cyp1a1-Fluc* mice for therapeutic development: 1) the peak of luminescence emitted following IP injection is much broader using the fungal system than the Fluc reporter, potentially allowing quick changes in AHR activity to be visualised in real time; 2) due to the distinct green-shifted emission spectrum and/or the distinct substrate requirements of nnLuz_v3, *Cyp1a1-nnLuz_v3* mice can be crossed with other bioluminescent models to examine tumour growth, infiltrates of specific cell types or expression of other genes, in tandem with AHR activity; and 3) following the optimisation of the luciferin biosynthesis pathway, *Cyp1a1-nnLuz_v3* mice have the potential to be adapted to become autoluminescent reporters of AHR activity.

In an animal model where a luciferase enzyme is used as a molecular reporter and the luciferin substrate is produced constitutively, obtaining real-time physiological information could be as simple as taking a photo in the dark. Current bioluminescent models (reviewed in [3,4]) would become less invasive, and the potential to split the pathway across cells or species could be harnessed to investigate host–microbe interactions (exemplified in Supplementary Figure S4). The *Neonothopanus nambi* bioluminescence pathway could be the best tool to generate such a mouse. There has been significant progress in adapting this pathway to produce autoluminescent plants and, unlike the bacterial system [24], there is no toxicity associated with pathway intermediates. However, progress in mammalian models was hindered by the temperature sensitivity of the luciferase enzyme [6]. Here, we showed that a thermostable variant of the fungal luciferase nnLuz could be used as a sensitive and reliable molecular reporter in vitro, in vivo and ex vivo, producing signal strong enough to be visualised on consumer grade cameras. Encouragingly, in mammalian cells, the transgenic expression of all members of the fungal bioluminescence pathway resulted in measurable luminescence. However, radiance was very low and not compatible with the high signal required for in vivo imaging. Therefore, the pathway needs to be further optimised before progression to in vivo models. Our results suggest that hispidin synthase is the rate-limiting step, and therefore boosting *HispS* expression is essential. This could be achieved by expressing *HispS* under the control of a strong constitutive promoter or incorporating multiple copies of *HispS* controlled by different promoters, an approach that could be fortified by diversifying the coding sequences of these *HispS* copies or using *HispS* orthologues from other fungal species.

In conclusion, we described a novel tool for visualising AHR activity whilst validating the use of the fungal luciferase nnLuz_v3 for in vivo imaging in warm-blooded animals, providing a promising and important step towards generating mammalian models with self-sustained fungal bioluminescence. *Cyp1a1-nnLuz_v3* mice were healthy, showing a strong bioluminescence signal upon AHR activation. The distribution and intensity of luminescence were comparable between *Cyp1a1-nnLuz_v3* mice and previously described *Cyp1a1-Fluc* animals [25], with the fungal luciferase offering some distinct advantages over Fluc that could be exploited for future AHR research.

## 4. Methods

### 4.1. Animal Maintenance

Mice were housed in a pathogen-free facility under the following conditions: 21 ± 2 °C; 45–65% humidity; 12 h light–dark cycle with RM3 diet and water ad libidum. Tissues, wood blocks, and tunnels were used to enrich the environment. All animal procedures were performed under a UK Home Office Project Licence and Personal Licences in accordance with the British Home Office Animal (Scientific Procedures) Act 1986 and approved by the Imperial College ethics committee (AWERB: Animal Welfare and Ethical Review Body). Mice were housed and experiments performed at Imperial College, which holds a UK Home Office Establishment Licence (X32FDCFC1), first approved 28.04.87.

### 4.2. Mammalian Cell Culture and Treatment

ES-E14TG2a (E14) mESC line (ATCC No.: CRL-1821) was used for gene targeting experiments to generate knock-in mESCs clones. mESCs were cultured on a layer of mitotically inactivated mouse embryonic fibroblasts on 0.1% gelatin-coated dishes with KnockOut Dulbecco’s Modified Eagle’s Medium (Gibco, Thermo Fisher Scientific, Loughborough, UK), supplemented with 15% foetal bovine serum (Gibco), 0.5% penicillin–streptomycin (Gibco), 0.1 mM non-essential amino acids (Gibco), 2 mM L-glutamine (Gibco), 0.1 mM 2-mercaptoetanol (Sigma, St. Louis, MO, USA) and 103 U/ml of leukaemia inhibitory factor (ESGRO, Millipore, Watford, UK). Cells were incubated at 37 °C with 5% CO2 and split every 2–3 days. Plasmid DNA transfections on mESCs were performed using Lipofectamine 2000 (Invitrogen, Paisley, UK) and Opti-MEM (Gibco), with cells in suspension in medium without penicillin–streptomycin, following manufacturer instructions. mESCs were treated with 10 nM FICZ (BML-GR206-0100, Enzo, Exeter, UK) or DMSO (Sigma) as vehicle control.

### 4.3. Cloning, CRISPR and Generation of Cyp1a1-nnLuz_v3 mESCs and Mouse Lines

The *nnLuz_v3* gene sequence was provided by Light Bio and codon-optimised for expression in mammalian cells. Gene targeting of *nnLuz_v3-T2A-iRFP670* reporter into mouse *Cyp1a1* gene was performed using CRISPR in mESCs E14 with an sgRNA sequence (5′- TCTTCAGGCTTAGACTGTCC -3′) specific to cleave DNA 4 nucleotides downstream of the stop codon located in exon 7. The sgRNA sequence and its corresponding antisense complementary sequence were ordered as DNA oligos (Sigma) and cloned using BbsI restriction enzyme (Thermo Fisher Scientific, Loughborough, UK) into the Cas9-GFP-containing plasmid pX458. pSpCas9(BB)-2A-GFP (pX458) was a gift from Feng Zhang (Addgene plasmid # 48138; chttp://n2t.net/addgene:48138, URL (accessed on 20 October 2026).; RRID: Addgene_48138) [26]. To induce reporter knock-in integration by DNA repair through homologous recombination, a donor DNA template containing 1 Kb of each homology arm was generated and used as a targeting vector. We also included a modified version of the internal ribosomal entry site sequence (IRES2) between the stop codon of *Cyp1a1* and the start codon of *nnLuz_v3* to allow independent protein synthesis of the reporter nnLuz_v3-T2A-iRFP670 polypeptide from the endogenous Cyp1a1 protein. In addition, a neomycin resistance cassette (*NeoR*) flanked by loxP sites was included in the donor DNA template for selection of mESC clones. Each component of the targeting vector was generated by PCR from existing DNA plasmids or, in the case of the homology arms, from genomic DNA from E14 mESCs. After purification, PCR fragments were cloned into the targeting vector through Gibson assembly method using a NEBuilder HiFi DNA Assembly Cloning Kit (E5520S, NEB, Hitchin, UK) according to manufacturer instructions. CRISPR was performed on E14 mESCs by co-transfecting both pX458 and targeting control plasmids using Lipofectamine 2000 (Invitrogen), followed by FACS enrichment of GFP-positive cells 24 h post-transfection. GFP-positive mESCs were seeded at very low density in 10 cm plates containing a layer of mitotically inactivated mouse embryonic fibroblasts on 0.1% gelatin-coated dishes. After 1 week, mESC colonies were manually picked under the microscope and individually expanded in the presence of neomycin (150 μg/ml). Individual clones were screened by PCR and selected based on phenotypic similarities to parental non-transfected E14 mESCs, such as growth rate and cell morphology. Karyotype was examined on selected clones prior to microinjection into blastocysts for mouse line generation. Primers used for *Cyp1a1-nnLuz* mESCs and mice genotyping are outlined in Table 1.

### 4.4. Other Animal Lines

*Cyp1a1-Fluc* mice were generated by OzGene (Bentley DC, Australia). A firefly luciferase (Fluc) gene was inserted into endogenous *Cyp1a1*, separated from the C-terminal region by a T2A sequence (see [15] for details).

### 4.5. In Vivo Experiments

For all in vivo experiments involving *Cyp1a1-Fluc* or *Cyp1a1-nnLuz_v3* animals, adult (6- to 12-week-old) mice were first weighed and intraperitoneal (IP) injected with 3-mC (213942, Sigma) dissolved in corn oil (Sigma) at 26.5 mg/kg. Corn oil was used as a vehicle control. Where possible, cage-mate siblings were used for test and vehicle measurements to minimise potential confounders. No animals had been subject to any additional procedures. Animals were chosen at random for 3-mC or vehicle injection; however, blinding was not performed to ensure consistency in the layout of the bioluminescence images produced (with control mice at the side of the image). Criteria dictated a priori stated that animals be excluded from experiments if in visible poor health (abnormal weight, activity or coat condition) at onset or should health deteriorate following 3-mC injection. No animals were excluded from the experiments or analysis.

### 4.6. Bioluminescence Imaging

All bioluminescence imaging was performed using IVIS Spectrum (Perkin Elmer, High Wycombe, UK) and Living Image software (version 4.3.1), using a stage temperature of 37 °C.

Fungal luciferin was synthetised as described in [27]. For imaging experiments, luciferin was dissolved in DMSO to a concentration of 200 mg/ml, then diluted in dH_2_O to working concentrations on the day of use. D-luciferin (Perkin Elmer) was dissolved and stored in dH_2_O.

For bioluminescence imaging of mESCs, fungal luciferin was diluted in mESC medium to achieve a final concentration of 650 µg/ml. Cells were imaged with IVIS two minutes after exposure to fungal luciferin using field of view C, binning 4 and depth 0.5 for a total of 5 minutes.

For in vivo bioluminescent imaging experiments, *Cyp1a1-Fluc* or *Cyp1a1-nnLuz_v3* adult mice were IP injected with 3-mC 5 h (IP) or ~14 h (oral gavage) before luciferin exposure. Mice were IP injected with 0.15 mg/g D-luciferin (*Cyp1a1-Fluc*) or 0.15 mg/g fungal luciferin (*Cyp1a1-nnLuz*). For oral gavage, *Cyp1a1-nnLuz_v3* adult mice were fed 0.15 mg/g fungal luciferin. Mice were anesthetised by isoflurane inhalation and imaged using IVIS Spectrum within 10–15 min of luciferin exposure. When assessing the kinetics of luminescence emitted from *Cyp1a1-Fluc* and *Cyp1a1-nnLuz_v3* mice, consecutive images were taken across a 90–120 min period, and the animals remained anesthetised throughout. Half-life values were calculated using the formula t_1/2_ = 0.693/ K_el_, where K_el_ is the absolute value of the SLOPE with y values converted to natural log.

Adult mice were IVIS imaged using 30 s exposure, field of view C or D, binning 1 and depth 1.5. In the majority of cases, the ‘open’ filter was used, capturing all emitted light. For generation of emission spectra, consecutive images were taken on IVIS Spectrum using emission filters with wavelengths ranging from 500 to 700 nm with 20 nm bandpass.

### 4.7. RNA Extraction and RT-qPCR

RNA was extracted using an RNeasy Mini Kit (Qiagen, Manchester, UK). Tissue samples were lysed in RLT buffer with a 5 mm stainless steel bead (Qiagen) using a TissueLyser II (Qiagen) for 4 min at 24,000 rpm. To digest fibrous tissue, heart samples were incubated with 10 μg/ml Proteinase K at 55 °C for 1 h. Next, to remove debris, all tissue samples were centrifuged at top speed for 3 min and the supernatant retained. Total RNA was purified from the supernatant using an RNeasy Mini Kit according to the manufacturer’s instructions, including the on-column DNase digestion step using an On-column RNase-Free DNase Set (Qiagen).

RT-qPCR was performed using QuantiTect SYBR Green PCR mix (Qiagen) in a CFX thermocycler (Bio-Rad, Hemel Hempstead, UK) with 0.4 μM primers (sequences in Table 1). The amplification protocol consisted of 40 cycles of 94 °C for 15 s and 60 °C for 30 s. For each set of primers, efficiency was calculated using the formula E  =  10 − 1/Slope. Relative RNA levels were then determined using the formula EI^control Ct-sample Ct^/EH^control Ct-sample Ct^, where EI  =  efficiency of primers of interest, and EH  =  efficiency of the housekeeping primer set, *Tbp*.

### 4.8. Autonomous Bioluminescence in HEK293T Cells

#### 4.8.1. Exogenous Substrate Addition

HEK293T cells (ATCC No.: CRL-3216) were seeded into Sarstedt Lumox 96-well plates at appr. 10,000 cells per well in DMEM (PanEco, Moscow, Russia) cultural medium supplemented with 10% FBS. Then, 24 h after seeding, cells were transfected with plasmid mixture (Table 2) by PolyFect (Qiagen) transfection reagent (1.5 µL per well); 24 h after transfection, growth medium was removed, and PBS with or without luminescence pathway substrate was added (all at 100 µM). The luminescent signal was registered by Tecan Spark plate reader during 30 min (48 time frames), 1 s per well exposure.

#### 4.8.2. Gene Titration

HEK293T cells were seeded into Sarstedt Lumox 96-well plates at appr. 10’000 cells per well in DMEM (PanEco) cultural medium supplemented with 10% FBS. Then, 24 h after seeding≤ cells were transfected with plasmid mixture (Table 3) by PolyFect (Qiagen) transfection reagent (1 µL per well); 24 h after transfection, growth medium was removed, and PBS with 100 µM of caffeic acid was added. The luminescent signal was registered immediately by Tecan Spark plate reader during 20 min (33 time frames for decreasing gene titration and 41 time frames for increasing gene titration), 1 s per well exposure.

### 4.9. Statistics and Reproducibility

Microsoft Excel was used for calculations, and GraphPad Prism (version 10) was used to create graphs and for statistical analysis. Graphs show the mean of experimental replicates, with error bars showing standard error (SEM). Multi-group comparisons were tested using ANOVAs with Dunnett’s or Šidàk’s multiple comparison tests. Specific details of each test are provided in the figure legends.

## Figures and Tables

**Table 1:** Primer and oligo sequences. F, forward; R, reverse; RevCom, reverse complement. Underlined sequences indicate *BbsI* site used for cloning and transcription of sgRNA in pX458 vector. UTR, untranslated region.

Assay	Purpose	Target	Sequence (5′ to 3′)
qPCR	Genotyping	*nnLuz_v3* (F)	CTGATCGACAAAGTGATCCC
qPCR	Genotyping	*nnLuz_v3* (R)	CTGGGGATGTTCAGAGGG
qPCR	Genotyping	*iRFP670* (F)	AGGTCGATCTCACCTCCTGC
qPCR	Genotyping	*iRFP670* (R)	CCGGCATTTTCCGTAATGC
qPCR	Genotyping	*Cyp1a1* (F)	CTGTGAACACTTCCAAGTGC
qPCR	Genotyping	*Cyp1a1* (R)	TGTGCCCAGTGTGTGTTCAG
qPCR	Genotyping	*NeoR* (F)	TGATTGACAAGACCGGCTTCC
qPCR	Genotyping	*NeoR* (R)	CTGTCATCTCACCTTGCTCC
RT-qPCR	Gene Expression	*Cyp1a1* (F)	CCTGTCCTCCGTTACCTGCC
RT-qPCR	Gene Expression	*Cyp1a1* (R)	AGGCTGTCTGTGATGTCCCG
RT-qPCR	Gene Expression	*Cyp1b1* (F)	CCACCAGCCTTAGTGCAGAC
RT-qPCR	Gene Expression	*Cyp1b1* (R)	GGCCAGGACGGAGAAGAGT
RT-qPCR	Gene Expression	*Tbp* (F)	GAAGAACAATCCAGACTAGCAGCA
RT-qPCR	Gene Expression	*Tbp* (R)	CCTTATAGGGAACTTCACATCACAG
CRISPR	Gene targeting	*Cyp1a1* 3′ UTR	CACCGTCTTCAGGCTTAGACTGTCC
CRISPR	Gene targeting	*Cyp1a1* 3′ UTR (RevCom)	AAACGGACAGTCTAAGCCTGAAGAC

**Table 2:** Autonomous bioluminescence HEK293T cell plasmid mixture.

Plasmid	Link	Amount per Well
*pCMV - HpaC - 3′UTR_SV40*	https://benchling.com/s/seq-y8xzpxq9TLLVO5Ixpga1	25 ng
*pCMV - HpaB - 3′UTR_SV40*	https://benchling.com/s/seq-xHOdW4VfdL5RKY8f1afX	25 ng
*pCMV - RcTAL - 3′UTR_SV40*	https://benchling.com/s/seq-EALzCyyBM2CVLEHFTzuG	25 ng
*pCMV - nnLuz_v4 - tSV40*	https://benchling.com/s/seq-43dTNxDtZPOiOh2OKyKe	25 ng
*pCMV - nnH3H_v2 - tSV40*	https://benchling.com/s/seq-CtGTqXBypyVV6gkO1rrj	25 ng
*pCMV - nnCPH - tSV40*	https://benchling.com/s/seq-d56KUa9vSH2fBpYT4aSF	25 ng
*pCMV - SFP - tSV40*	https://benchling.com/s/seq-aK15X8T4vDqR5R090gov	25 ng
*pCMV - mcitHispS - tSV40*	https://benchling.com/s/seq-yiQg45S3X9Lzxv0oQHSz	50 ng

URLs accessed on 20 October 2026.

**Table 3:** Gene titration plasmid mixture.

Plasmid	Link	Amount per Well
*pCMV - nnLuz_v4 - tSV40*	https://benchling.com/s/seq-43dTNxDtZPOiOh2OKyKe	25 ng (2.5 ng in low *nnLuz*)
*pCMV - nnH3H_v2 - tSV40*	https://benchling.com/s/seq-CtGTqXBypyVV6gkO1rrj	25 ng (2.5 ng in low *H3H*)
*pCMV - nnCPH - tSV40*	https://benchling.com/s/seq-d56KUa9vSH2fBpYT4aSF	25 ng
*pCMV - SFP - tSV40*	https://benchling.com/s/seq-aK15X8T4vDqR5R090gov	25 ng (2.5 ng in low *SFP*)
*pCMV - mcitHispS - tSV40*	https://benchling.com/s/seq-yiQg45S3X9Lzxv0oQHSz	50 ng (5 ng in low *HispS*)

URLs accessed on 20 October 2026.

## Data Availability

All numerical source data are provided in Supplementary Data File S1.

## Supplementary Materials

The following supporting information can be downloaded at: https://www.mdpi.com/article/10.3390/ijms27219586/s1. Refs. [17,25] are cited in Supplementary Material.
