# Supplementary material for: Quantitative Bioluminescent Imaging of AHR Activity Using a Thermostable Variant of the Fungal Luciferase nnLuz
Source: Int J Mol Sci. 2026 Oct 27;27(21):9586. doi: 10.3390/ijms27219586 (PMC13762613; doi:10.3390/ijms27219586)
Supplement: Supplementary file 1 [file ijms-27-09586-s001.zip › ijms-4635971-supplementary-Figures.pdf]

Supplementary Figures

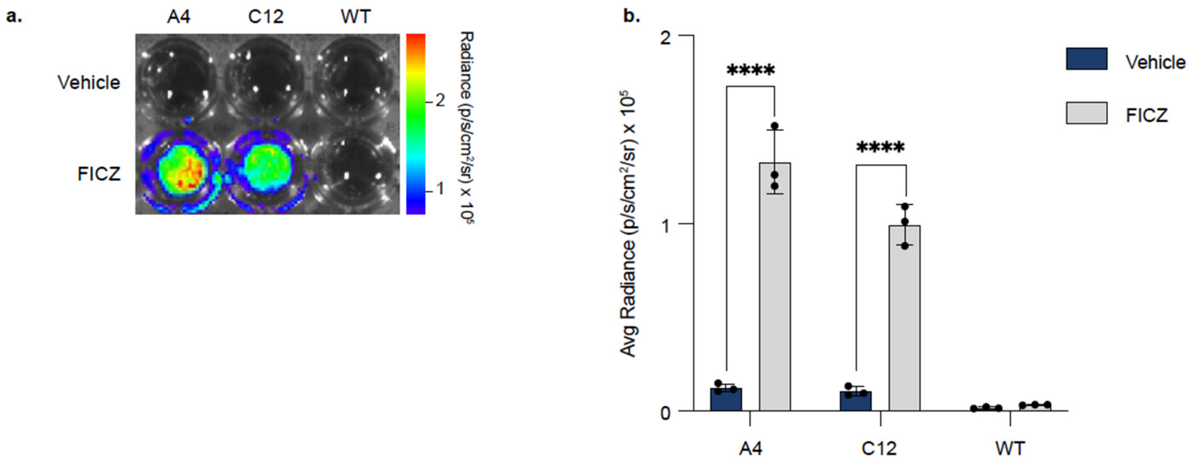

Supplementary Figure S1: Bioluminescent images of *Cyp1a1-nnLuz_v3* mESCs.

Cyp1a1-nnLuz_v3 mESC clones were exposed to FICZ or vehicle for 4 h. Representative images (**a**) and quantification (average radiance) (**b**) of bioluminescent signal after incubation with fungal luciferin (650 µg/ml)**.** Two-way ANOVA with Šidák's multiple comparison test to compare vehicle to luciferin treated samples, **** $p<0.0001$. Bars show mean (n=3) +/- SEM.

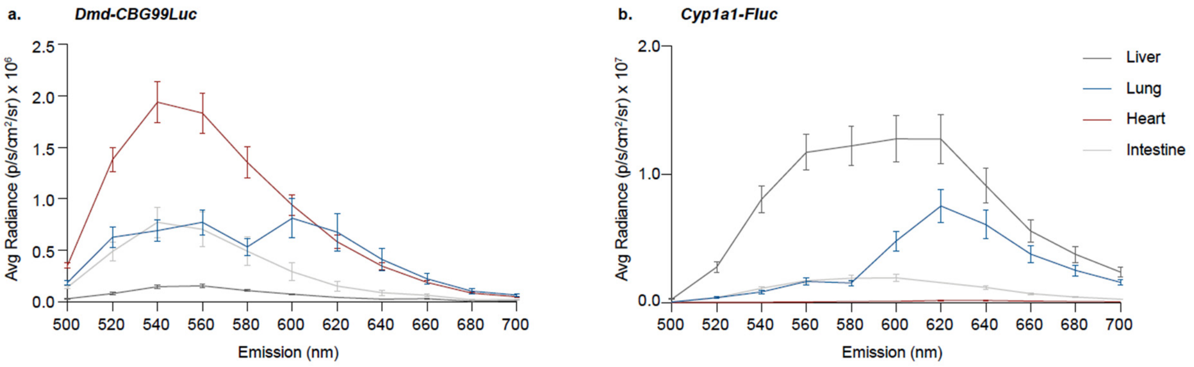

Supplementary Figure S2: Tissue specific emission spectra of *Cyp1a1-Fluc* and *Dmd-CBG99Luc* bioluminescent reporter mice.

Tissues were dissected from *Dmd-CBG99Luc* (**a**) and *Cyp1a1-Fluc* (**b**) mice after IP injection with 0.15 mg/g D-luciferin. Bioluminescence imaging performed using 20 nm band pass emission filters across a 500–700 nm range. Graphs show quantification of average radiance (n=4 for (**a**) and n=3 for (**b**)). **b.** Mice were injected with 3mC 5 h prior to luciferin exposure. For **a.** data is taken from [17].

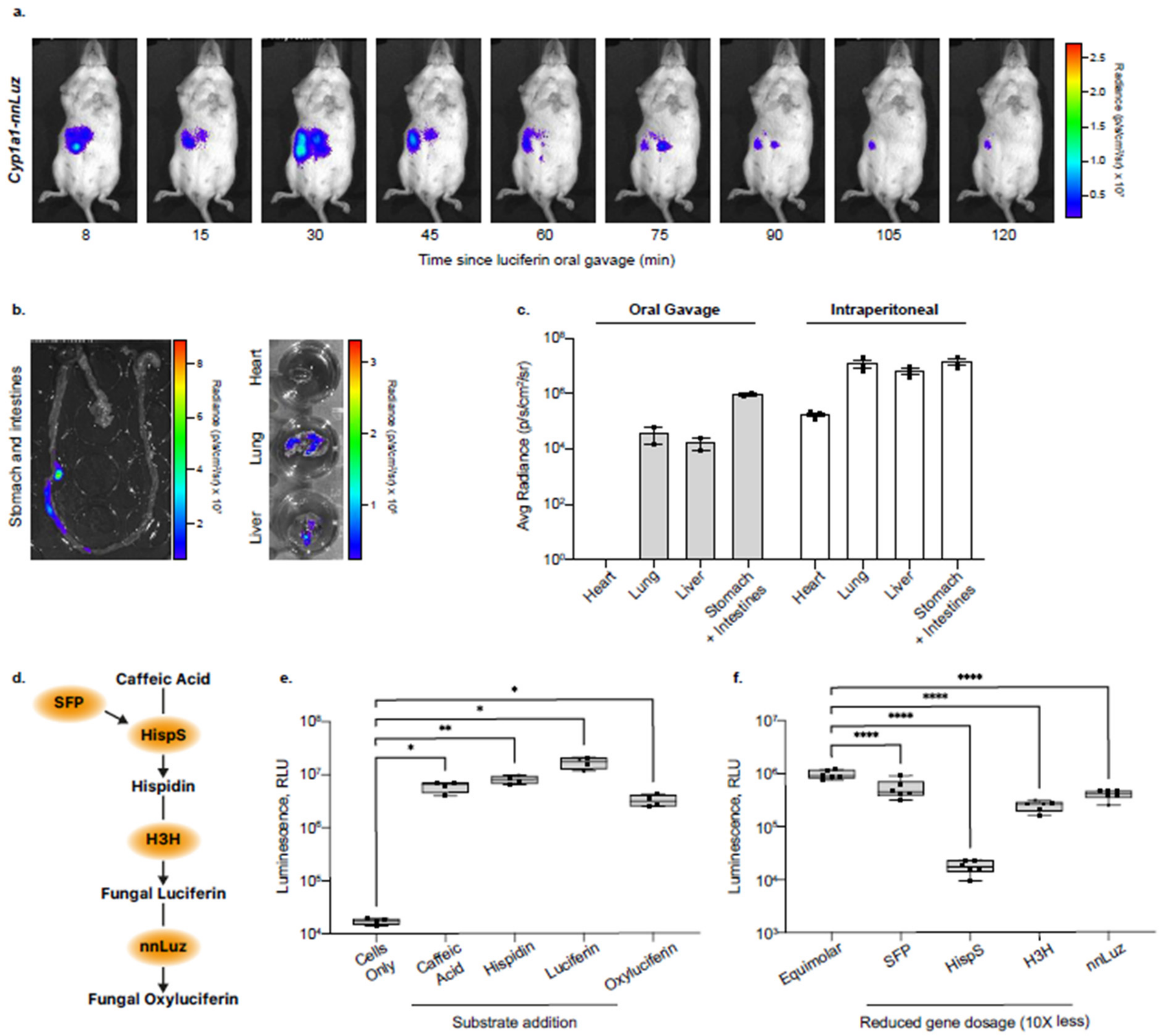

Supplementary Figure S3: Oral availability of fungal luciferin.

a. *Cyp1a1-nnLuz_v3* mice were fed 0.15 mg/g fungal luciferin by oral gavage. Mice were IP injected with 3mC the night before imaging. Mice were repeatedly imaged across a 120-minute period post oral gavage. Representative bioluminescence images are shown. **b.** *Cyp1a1-nnLuz_v3* mice were fed 0.15 mg/g fungal luciferin by oral gavage. Mice were IP injected with 3mC the night before imaging. Bioluminescence imaging was performed on tissues dissected from the mice in **Figure 2 g**. Representative images of tissues. **c.** Quantification of radiance emitted from *Cyp1a1-nnLuz_v3* tissues following exposure to 0.15 mg/g fungal luciferin by oral gavage (representative images in **b**) or intraperitoneal injection (representative images in **Figure 2 c**). Bars show average radiance minus background. Shown in $\log_{10}$ scale, mean (n= 2 for oral gavage and n=3 for IP injection) +/- SEM. **d.** Schematic representing the enzymes and intermediates in the fungal bioluminescence pathway. **e.** HEK293T cells were transfected with plasmids corresponding to all enzymes in the fungal bioluminescence pathway. 24 h after transfection cells were incubated with vehicle (cells only), Caffeic acid, Hispidiin, Luciferin or Oxyluciferin at 100 mM and imaged on a Tecan Spark plate reader. Boxes

show 25th to 75th percentiles, with central line representing the median and whiskers showing the full range of values (n=4). A one-way ANOVA compared ‘cells only’ with substrate added wells (*p<0.05, **p<0.01). **f.** HEK293T cells were transfected with enzymes of the fungal bioluminescence pathway at equimolar concentrations, or with individual genes (*SFP, HispS, H3H* or *nnLuz*) at a concentration reduced by 10-fold. Box and whiskers plots represent data as of (**g**) (n=6). A one-way ANOVA compared equimolar with gene depleted wells (****p<0.0001).

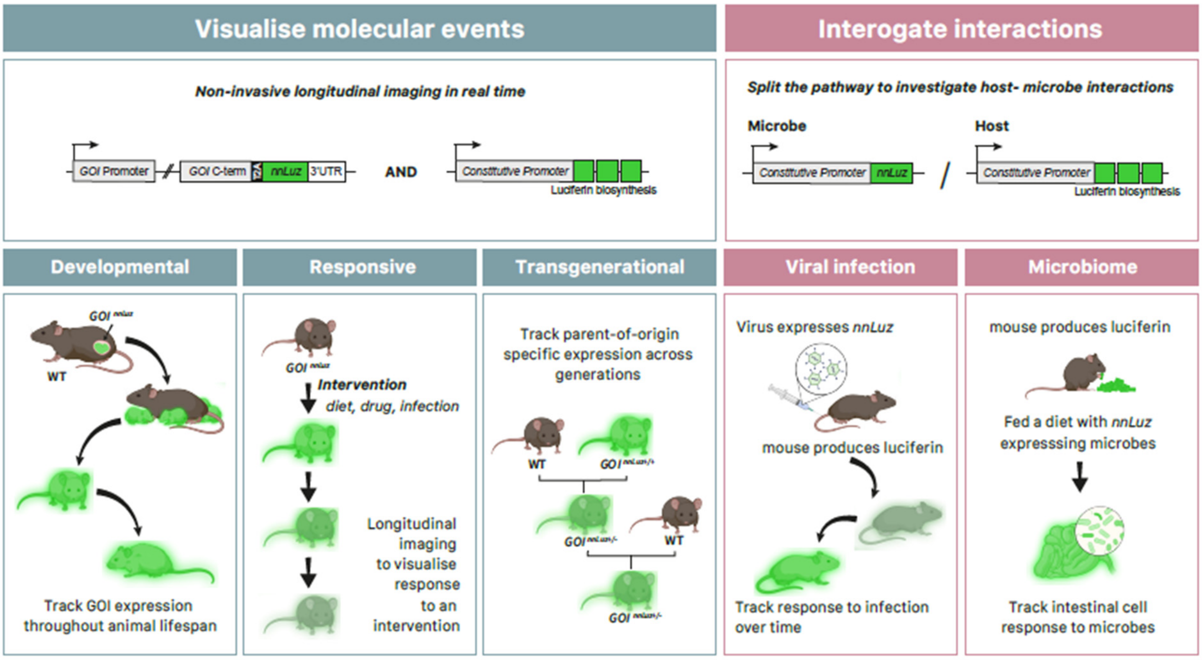

Supplementary Figure S4: Biosynthesis of fugal luciferin and the future potential of autoluminescent mammalian models

a. Schematic representing some of the potential uses of animal models expressing the fungal luciferase/luciferin pathway. **Visualise molecular events:** The first panels (blue) show the use of the fungal pathway to generate molecular markers. The luciferase can be inserted into an endogenous gene of interest (GOI), while the luciferin is constitutively and ubiquitously expressed in the same animal. Therefore, the animal will luminescence whenever the GOI is expressed. This can be harnessed to improve developmental, responsive and transgenerational studies. **Developmental**: Non-invasive imaging allows for GOI expression to be tracked in the same animal across the entire lifespan, from in utero [27] to old age. **Responsive**: Expression of a GOI in response to an external intervention such as drug treatment, diet, environment, illness or infection. Animals can be imaged repeatedly following intervention. **Transgenerational**: Parent-of-origin specific expression can be tracked across generations, and in response to interventions, to investigate imprinting, X-linked or mitochondrial disorders. **Investigate interactions:** Second panels (pink) show the potential to split the pathway across organisms. Here a host animal would express the genes required for luciferin biosynthesis and a microbial species would express the luciferase, *nnLuz*. **Viral infection**: The virus expresses the luciferase and the mouse produces the luciferin. Non-invasive imaging could allow you to track progress of the infection in real time. **Microbiome:** Introduce *nnLuz* expressing microbes into the microbiome of a luciferin producing mouse. The tissue specific response to the microbes could be tracked. Some elements of this figure were generated using Biorender.com.
